# Biotic Interactions in Soil are Underestimated Drivers of Microbial Carbon Use Efficiency

**DOI:** 10.1007/s00284-022-02979-2

**Published:** 2022-12-02

**Authors:** Hélène Iven, Tom W. N. Walker, Mark Anthony

**Affiliations:** 1 Department of Environmental Systems Science, Institute of Agricultural Sciences, ETH Zurich, 8006 Zurich, Switzerland; 2 Institute of Biology, University of Neuchâtel, 2000 Neuchâtel, Switzerland; 3 Department of Environmental Systems Science, Institute of Integrative Biology, ETH Zürich, 8006 Zurich, Switzerland

## Abstract

Microbial carbon use efficiency (CUE)—the balance between microbial growth and respiration—strongly impacts microbial mediated soil carbon storage and is sensitive to many well-studied abiotic environmental factors. However, surprisingly, little work has examined how biotic interactions in soil may impact CUE. Here, we review the theoretical and empirical lines of evidence exploring how biotic interactions affect CUE through the lens of life history strategies. Fundamentally, the CUE of a microbial population is constrained by population density and carrying capacity, which, when reached, causes species to grow more quickly and less efficiently. When microbes engage in interspecific competition, they accelerate growth rates to acquire limited resources and release secondary chemicals toxic to competitors. Such processes are not anabolic and thus constrain CUE. In turn, antagonists may activate one of a number of stress responses that also do not involve biomass production, potentially further reducing CUE. In contrast, facilitation can increase CUE by expanding species realized niches, mitigating environmental stress and reducing production costs of extracellular enzymes. Microbial interactions at higher trophic levels also influence CUE. For instance, predation on microbes can positively or negatively impact CUE by changing microbial density and the outcomes of interspecific competition. Finally, we discuss how plants select for more or less efficient microbes under different contexts. In short, this review demonstrates the potential for biotic interactions to be a strong regulator of microbial CUE and additionally provides a blueprint for future research to address key knowledge gaps of ecological and applied importance for carbon sequestration.

## Introduction

Soil microbes are major actors in the terrestrial carbon cycle [1]. Microbial products (e.g. necromass, proteins, DNA) commonly comprise 10–80% of the total soil organic carbon (SOC) stock [2], and the formation and stabilization of these products are a key determinant of ecosystem carbon sequestration. At the same time, microbial activity accounts for approximately 60% of global soil carbon dioxide (CO_2_) emissions, making microbes an important component of the terrestrial carbon balance [3]. Two fundamental processes influence microbial SOC formation and depletion: growth, which produces biomass that may eventually become SOC; and respiration, which releases SOC as CO_2_. The balance between microbial respiration and growth is termed microbial carbon use efficiency (CUE; a.k.a. growth efficiency or yield), and is specifically defined as the proportion of assimilated carbon used for building new biomass relative to that lost through respiration and the activity of endogenous metabolism [4, 5]. CUE is one of the few explicit microbial variables in SOC cycling models [6], so accurately predicting it is therefore of considerable interest. Yet, our ability to do so is limited by an incomplete understanding of the factors affecting CUE. Here, we argue that biotic variables, such as competition and facilitation, constrain CUE above and beyond abiotic controls and should be explicitly included in the next phase of CUE research. While the effects of abiotic factors on CUE have been extensively tested, e.g. in [7], much less is known about how biotic factors, such as competition, facilitation, predation, and plant–microbe interactions, affect CUE.

This review aims to guide researchers towards a coherent research direction by linking microbial biotic interactions to microbial physiology with implications for SOC cycling. We draw from multiple lines of theoretical and empirical evidence from the evolution and botanical literature, which collectively suggest that biotic interactions should strongly affect CUE through differences in life history strategies. The most relevant life history frameworks include *K-* vs. *r*- selection [8], the competition-stress-ruderal (C-S-R) life history axes [9], and a reimagined microbial C-S-R that considers growth yield (Y), resource acquisition (A), and stress tolerance (S)—referred to as the Y-A-S framework [10]. While all of these life history frameworks directly or indirectly consider biotic interactions in the context of competition for resources, we argue that greater development and additional explicit biotic life history traits could be integrated into these frameworks to better predict CUE. CUE is an emergent property of multiple abiotic and biotic factors that are difficult to disentangle. However, by isolating the contributing effects of specific biotic interactions on CUE, it should be possible to disentangle the mechanisms by which microbes respond to abiotic changes. Doing so could thus allow us to predict microbial physiological performance under changing and/or novel environmental conditions. There is a wide range of biotic interactions occurring among soil microbes, including competition, predation, facilitation, and mutualisms among microbes and with higher trophic level organisms [11]. Hence, CUE may be impacted by many different types of biotic interactions simultaneously. Because microbiomes are typically hyper-diverse in the sense of diversity and functionality, the combined sum of positive (facilitation), neutral (commensal) and negative interactions (competition) will drive CUE at the aggregate community level [12]. Competition relates to negative interactions that deplete a population through the activity of antagonists onto protagonists [13]. It can occur among the same species (intraspecific competition) or among different species (interspecific competition), and can be direct (i.e. interference), indirect (i.e. exploitative), or predator-mediated [14]—though these forms of competition often overlap among microbes [15, 16]. Because competitive interactions commonly require life history strategies that promote fast growth and extensive investments in resource acquisition by antagonists and stress response by protagonists, we hypothesise that competition causes microbes to grow less efficiently. Facilitation relates to myriad positive interactions between organisms that benefit at least one organism and cause no harm to either organism [12, 13]. Facilitation can favour high CUE by (1) ameliorating abiotic stress; (2) creating novel habitats to promote niche partitioning; (3) increasing habitat complexity and heterogeneity; (4) sharing services like producing common goods and (5) increasing the availability of otherwise inaccessible resources [12]. Mutualism is a specialized form of facilitation that benefits both species, such as the exchange of services commonly observed between host plants and rhizobia or mycorrhizal fungi [13]. Because facilitation regularly promotes the exchange of resources and ameliorates stress, we anticipate that positive biotic interactions promote efficient microbial growth and may increase CUE at the community level if the sum of facilitation is greater than the combined sum of commensal plus competitive interactions.

To address these two expectations, we draw on theoretical and empirical lines of evidence to determine how biotic interactions (competition, predation, facilitation, and interactions with plants) drive certain microbial life history strategies, with a particular focus on implications for CUE and SOC cycling. While the focus of this review is on CUE, we also critically evaluate the current state of knowledge on microbial species interactions and life history characteristics relevant to CUE. First, we address intraspecific interactions and density-dependent feedbacks to provide a foundation for understanding relationships between microbial growth and biotic interactions (“CUE Fundamentally Depends On Density Dependence And Carrying Capacity”) section. We then discuss the roles of interspecific direct competition (“Interspecific Direct Competition Induces Metabolic Costs Leading to Low CUE”) section, interspecific indirect competition (“Interspecific Indirect Competition And Its Coincidence With Environmental Heterogeneity”) section, facilitation (“Facilitation Among Microbes Promotes Coexistence and Increases CUE”) section, predation (“Predation Influences Microbial Density and Competitive Outcomes”) section, and plants (“Effects of Plant Community and Plant-Microbe Interactions on CUE”) section on microbial CUE as well the influence of spatial separation among soil organisms (“Effect of Spatial Heterogeneity on CUE”) section—a unique but important characteristic of soil systems.

## Effects of Biotic Interactions on Microbial CUE

### CUE Fundamentally Depends on Density Dependence and Carrying Capacity

**Fig. 1:**
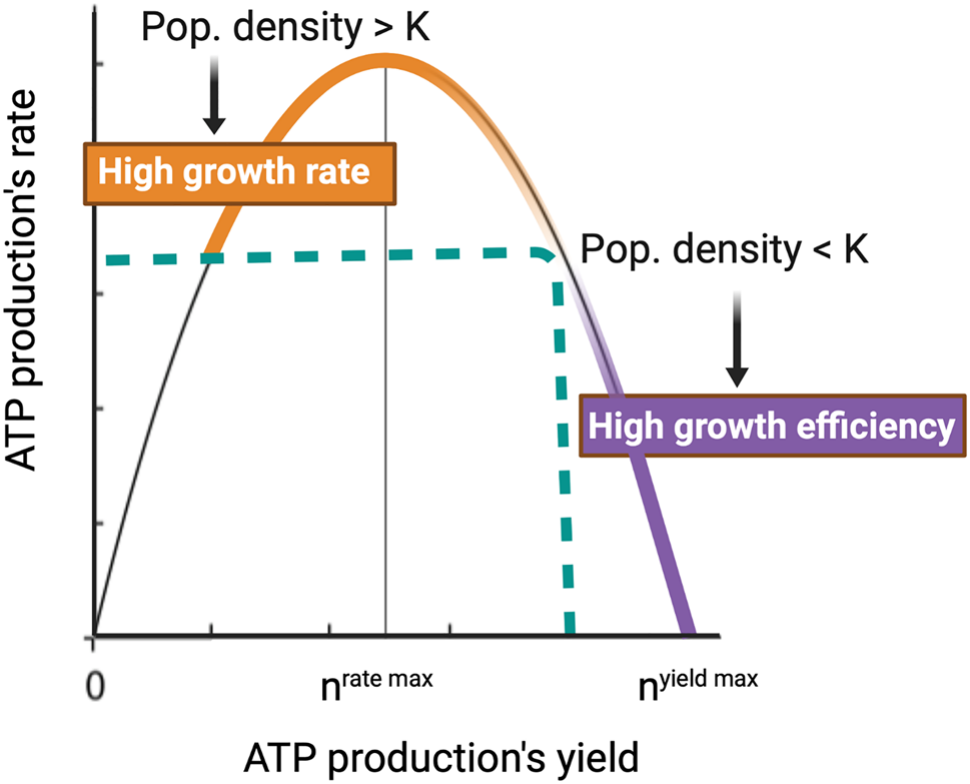
The tradeoff between high growth rate (yellow) and high CUE (purple, a.k.a. growth yield) has its origins in the thermodynamics of ATP production pathway, where the maximal rate of ATP production is obtained at half maximal ATP yield. In the absence of other biotic interactions than intraspecific competition, CUE depends on whether the population density is below or above carrying capacity (K). Figure modified from data in Pfeiffer and Bonhoeffer (2002) (Color figure online)

Density-dependent feedbacks on species abundances place important constraints on life history-related differences in CUE. Seminal eco-evolutionary work on microbial growth in culture has demonstrated that if the density of a microbial population is below its carrying capacity, then a slow but efficient metabolism should drive relatively low growth rates and relatively high CUE [17]. Because organisms cannot obtain maximal yield from a substrate at the maximal metabolic rate (Fig. 1); [18, 19], there is a tradeoff between growth efficiency and growth rate under certain environmental conditions [20], which is also reflected by the life history framework regarding r-/K-selection. The selective pressure on species to grow fast versus efficiently depends on whether a species typically reaches carrying capacity and in turn experiences intraspecific competitive exclusion [11, 18]. When cell cultures growing on a single carbon source reach high population densities, competition for shared resources intensifies and favours fast resource use, which is typically inefficient [17]. Evidence from culture studies thus raises numerous important questions regarding soil microbial growth—with an important first question being how often species in soil reach carrying capacity, if at all.

Carrying capacity is affected by eco-evolutionary forces, as well as by factors influencing how many individuals a habitat can support (*i.e.* habitat carrying capacity [19]). While individual microbial species carrying capacities are not well quantified in environmental habitats (*i.e.* soil, plants, animals), there is nonetheless sufficient evidence to indicate that they occur. For example, genetically labelled *Curvibacter* strains reach a carrying capacity of 2 × 10^5^ cells in their freshwater cnidarian host [21]. Microbial carrying capacities have also been estimated on plastic marine debris [22], absorptive versus transportive fine roots [23], and in the tissues and organs of different animals, such as zebrafish [24] and humans [25]. Furthermore, there is a wealth of studies relating to potting soil development that aim to increase microbial carrying capacities to provide biological control against plant pathogens (e.g. [26]). In order to resist *Rhizoctonia* pathogens, organic matter in potting mixes must become fully colonized by soil microorganisms, *i.e.* microbes must reach the habitat carrying capacity [27]. Thus, microorganisms can reach carrying capacity in environmental samples, including soil, but little is known about which environmental and eco-evolutionary forces influence where and which microbes do so.

We hypothesize that only certain soil microbes reach carrying capacity in nature because interspecific competition reduces individual species abundances [28], many species are dispersal limited [29], and, drawing on evidence from plant ecology, stochastic processes limit recruitment [30]. Thus, the probability of reaching carrying capacity may be generally low in natural soils and site-specific in terms of the taxa that are locally dominant. Observations that microbial communities are routinely highly uneven (i.e. extreme dominance by a small number of taxa [31]) suggest that only some of the most dominant species in a community may in fact reach carrying capacity, with the majority of less common taxa being too rare to do so. While many factors can generate uneven community distributions, species evenness and dominance have been used to infer carrying capacity dynamics among plants and animals (e.g. [32]). Further, dominant rather than rare bacteria in the human gut not only reach but exhibit similar carrying capacities across individuals even when overall community composition patterns vary [25]. A hypothesis for future study would thus be that when any dominant microbial species becomes more or less abundant as a consequence of carrying capacity, these taxa have disproportionately strong, yet potentially ephemeral, negative impacts on community-level CUE. While it is likely that species that become dominant in soil harbour certain traits, such as high stress tolerance and carbohydrate metabolism potentials among soil fungi [33] or high growth rates among bacteria (found in culture and soil [23, 34]), any organism that reaches carrying capacity should exhibit reduced CUE. Since microbial communities are routinely dominated by just a few taxa, it may be possible to isolate the growth dynamics of these species if they can be cultured or tracked in natural systems using isotopically labelled water or nutrients.

Another clear axis of importance when considering density dependence is identifying which resources are being metabolized and how they are distributed. Much experimental work on density dependence to date has been conducted using cultures growing on a single, non-limiting, carbon source, whereas in soil, there are many different substrates of varying qualities and quantities that are distributed heterogeneously. In general, microbes grow more efficiently on simple substrates, such as glucose, versus more complex substrates, such as plant residues with high carbon-to-nitrogen ratios (see [35] and [36] where this is discussed in detail). A large body of ecological models suggests that under heterogenous conditions, diffusing populations can reach a higher carrying capacity than under homogenous conditions [37, 38]. How species move in the soil and how heterogenous resources may be distributed could influence whether and to what degree species reach carrying capacity. Further, in the laboratory, yeast cells reach higher population sizes when growing on homogenously versus heterogeneously distributed resources [34]. How resource uniformity versus heterogeneity modulates the carrying capacity of soil microbes therefore remains an important area of future research and could be evaluated using microcosms consisting of patchily versus homogenously distributed resources or under fluctuating versus stable environmental conditions.

### Interspecific Direct Competition Induces Metabolic Costs Leading to Low CUE

The impact of direct competition on CUE depends on competitor density and the intensity of antagonistic interactions. Direct competition describes interactions between competitors to obtain space [14]. A clear example of this comes from a study of three different strains of *Escherichia coli*, which persist in spatially structured habitats that discourage direct competition but cannot co-exist in well-mixed environments as a result of competitive exclusion due to direct competition [11]. The acquisition of space can involve either the production of anti-microbial toxins or interference in the motility and signalling of competitors, which, in turn, alters the behaviour and physiology of both antagonists and protagonists, as seen in culture experiments [11]. For example, streptomycin released by species of the bacterial genus *Streptomyces* and phenazines produced by species of the bacterial genus *Pseudomonas* induce metabolic costs to antagonists [11]. In response, protagonists activate essential resistance mechanisms or warn related organisms through the production of volatile organic compounds, both of which impose endogenous metabolic costs to avoid cell damage and death [11]. Thus, high degrees of direct competition may decrease CUE of both protagonists and antagonists, at least in the short term, in comparison to another community in the same environment but without or under less direct competition.

Further experimental work has demonstrated that high competitor density forces microbes to adopt stress-tolerant life history strategies (e.g. by expressing phenotypes, such as sigma-factors or molecular chaperones). The best example of this is among saprotrophic fungi, which lower their CUE under direct competition [39]. When a protagonist is not resistant to an antagonist, the population of the protagonist can be depleted or even entirely replaced by the antagonist, thus altering the microbial community towards a highly competitive, less efficient community composition [40]. It remains unclear whether competitive dominance by antagonists (i.e. exclusion of the protagonist) feeds back to eventually alleviate antagonist investments in competition, allowing the antagonist to grow more efficiently in the context of increased resources due to protagonist exclusion. Nevertheless, protagonists that are particularly sensitive to competition tend to have lower metabolic costs than antagonists resistant to competition because they invest less in toxin production and toxin resistance mechanisms [40], suggesting a higher intrinsic CUE. In summary, antagonists, by negatively impacting sensitive taxa, inducing costly resistance mechanisms in resistant taxa, and by investing in toxin production themselves, decrease the overall CUE of a community (Fig. 1).

Building on general evidence that competition may reduce CUE, there remain a few key areas of uncertainty that future research should examine. One uncertainty is whether an antagonist that excludes a protagonist eventually alleviates investments in competitive strategies and increases its CUE. Tracking CUE over different stages of direct competition, such as prior to engagement, during chemical and physical interactions, and as one species dominates another, would provide new insight into this question. It is additionally unclear whether competition arising from specific contexts, such as low nitrogen availability, could help to select for more efficient species, alleviating competitive costs when compared to the same environment without competition. Beginning to disentangle these complex forms of interspecific competition will allow us to start predicting growth processes in soils inhabited by species known to engage in competition.

### Interspecific Indirect Competition and its Coincidence with Environmental Heterogeneity

**Fig. 2:**
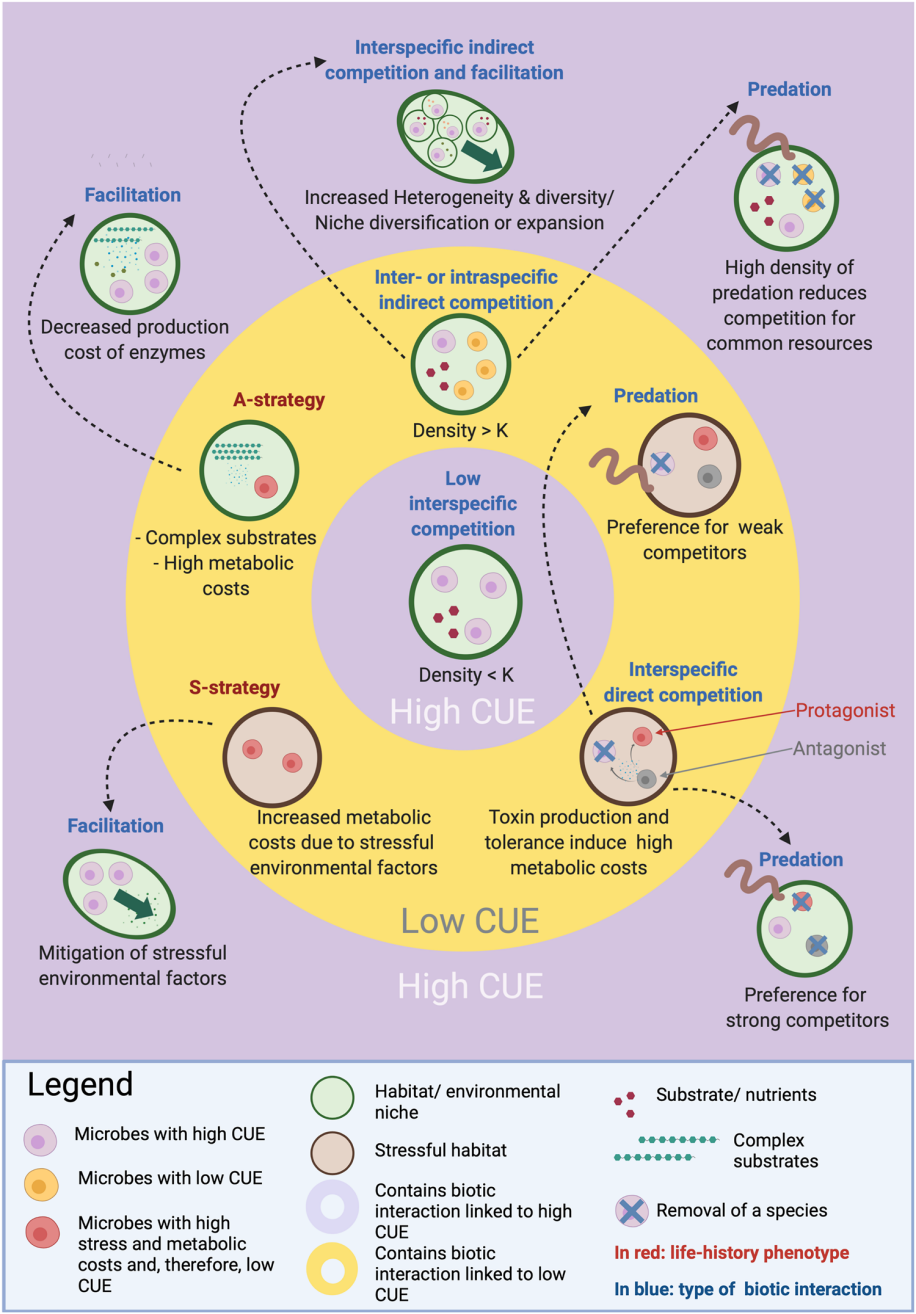
A summary of biotic interactions occurring in soil with implications for CUE. High CUE communities are characterized by facilitation, and, in specific contexts, interspecific competition and predation. Low CUE communities are characterized by high metabolic costs that do not generate growth (e.g. S- and A-strategy phenotypes), induce increases in growth rates (e.g. in response to direct competition), interspecific competition (in many instances), and during specific predator–prey interactions. Interspecific competition does not negatively impact CUE when population density is lower than carrying capacity (K). Green arrows represent an expansion of a species’ realized niche via facilitative effects. The black dashed arrows represent how a certain biotic interaction can change due to cascading effect of biotic interactions

Indirect competition impacts CUE by reducing resource availability. Indirect competition consists of competitors blocking or limiting access to resources [11]. This is critical because resource limitation in any form can reduce CUE (see review by [5]). When several species compete for the same resource, similarly to intraspecific competition, resource use must be faster and relatively less efficient than in the absence of competition [17]. However, in many natural environments, resources are patchily distributed in space and time. The degree of resource heterogeneity can also be enhanced by resource competition [41]. Patchy soil resource availability can lead to co-existence because the chances of taxa existing on the same spectrum of multiple fluctuating resources are rather low, as observed in computational experiments [41]. Ecosystem heterogeneity can therefore increase species co-existence by alleviating indirect competition, and this should lead to higher emergent CUE (Fig. 2).

Environmental heterogeneity can promote microbial niche partitioning and thereby reduce competition for common substrates [42, 43]. This mechanism was a pioneering conclusion of the “Paradox of the Plankton” proposed by [44], who observed high species diversity of plankton in lakes despite species possessing similar nutrient requirements. While lakes appear to be well-mixed environments, they are in fact composed of heterogenous micro-patches of nutrients, making them somewhat analogous to soil. Moreover, as species evolve, they develop different competitive abilities and specialize to decompose distinct substrates from their neighbours [45, 46]. It is important to acknowledge that there is still a considerable degree of functional redundancy among microbial communities [47, 48], but trait overlap does not necessarily translate to redundancies in the functioning of communities, as observed in freshwater ecosystems [49]. High levels of resource heterogeneity can also favour niche partitioning, which can promote diversity in soil by encouraging microbes to exit dormancy [45, 50]. This could increase CUE since microbial dormancy is generally believed to be inefficient [51]. The logic behind such a mechanism is well demonstrated, in that dormant microbes exhibit minimal anabolism but must invest in maintenance and repair costs [52]. Thus, for a number of reasons high environmental heterogeneity should promote high CUE.

One putative mechanism for a connection between environmental heterogeneity and CUE is high microbial diversity via niche partitioning. Recent evidence that microbial diversity corresponds to increases in CUE was documented by Domeignoz-Horta et al. [53], who assessed the direct effects of microbial diversity on CUE by establishing a microbial diversity gradient at two moisture levels and temperatures in an artificial soil environment. Adding cellobiose as a carbon source, they tracked CUE and correlated it with bacterial and fungal diversity. Bacterial phylogenetic diversity was positively correlated with CUE, but only under high soil moisture contents. These results suggest that, under high soil moisture, organisms may interact more cooperatively by sharing resources and that this is not possible when moisture limitations physically restrict microbial interactions. A key component of niche partitioning includes facilitative effects, and it would be interesting for future studies to consider how facilitation might vary with environmental conditions, including resource heterogeneity. While experimental evidence for diversity–CUE relationships is still rudimentary, recent experimental work not only suggests that microbial diversity is related to CUE, but also that positive species interactions (e.g. facilitation) may be an important underlying mechanism. Future work must now investigate the relationship between CUE and diversity using natural communities and under different abiotic conditions to identify specific facilitative mechanisms influencing CUE.

### Facilitation Among Microbes Promotes Co-existence and Increases CUE

Facilitation describes an interaction whereby the presence of one organism (the facilitator) benefits another (the facilitated) by improving its local environment [13]. Facilitation can increase CUE at population and community levels under certain circumstances in three ways: (1) increasing the size and the number of realized niches of facilitated species, thereby increasing local species richness; (2) mitigating stressful environmental conditions which constrain anabolism and (3) decreasing the production costs of extracellular enzymes. Perhaps most obviously, facilitators can enlarge facilitated species niches by directly improving the surrounding energy and nutrient contents required by the facilitated species [12, 13]. For instance, the release of microbial products, such as dead microbial cells or the remains of extracellular enzymes, can enlarge the realized niches of facilitated microbes by providing them with nutrient-rich microbial products, as observed in a computational model representing litter decomposition in soil [54]. This mechanism has been empirically shown in cultures using different populations of interacting *E. coli*, which can exhibit complementary metabolism by sharing hydrogen, acetate, amino acids, nitrogen and glucose, promoting the growth of each population [55]. While pervasive nutrient limitations are known to decrease CUE [5], a recent modelling study also demonstrated that co-existence increases with facilitative chemical interactions because microbial products, including metabolites and waste-products, provide limiting nutrients to other microbes [56]. Further, Kästner et al. [57] suggested in a recent review that microbes feeding on microbial necromass have a high CUE because necromass provides nutrients with similar stoichiometric ratios to living microbial biomass. Thus, resources can be recycled within a community, enlarging realized niches, and—if sufficiently widespread—increasing CUE at population and community levels.

Facilitative interactions can also promote survival during stressful abiotic conditions, such as drought [12]. Indeed, facilitation appears to be especially important in harsh and stressful conditions because it alleviates essential metabolic constraints on the facilitated species [13]. For instance, in a culture experiment, the presence of microbial species resistant to competition increased the survivorship of other microbial species less resistant to competition [58]. While the exact mechanism of such induced resistance remains unclear, horizontal transfer of genes conferring resistance to antibiotics has been repeatedly observed among bacteria in soil [59, 60] and is very likely to be one of a number of mechanisms by which resistance against anti-microbial compounds is obtained by otherwise sensitive species. Ultimately, when microbes are not forced to respond to stress, they can invest relatively more energy into growth (or other processes), and we therefore expect this to increase the CUE of facilitated species (Fig. 2).

Finally, facilitation can reduce the energetic costs required to break down complex substrates. For instance, a recent simulation study demonstrated that aquatic bacterial communities grow more efficiently by aggregating enzyme production at the community level [61]. In extreme cases, microbial products, such as enzymes or molecules that scavenge iron (siderophores), can lead to “cheaters”, which are organisms that realize the benefits of such facilitative interactions without producing extracellular enzymes or siderophores [62, 63]. Although the presence of cheaters may negatively affect some ecosystem processes, such as mycorrhizal nutrient transfer, they nevertheless increase total microbial community biomass relative to extracellular enzyme production, resulting in higher CUE at the community level [63]. However, when the density of non-cheating microbes exceeds a certain threshold, each individual receives less per capita resource, which leads to enhanced competition and subsequently promotes rapid growth rates, lowering community-level CUE [61, 62]. In short, when facilitative processes lower the costs of enzyme production for specific community members or the whole community without dramatically reducing the amount of resources per capita, the CUE of the community may increase.

### Predation Influences Microbial Density and Competitive Outcomes

Microbial predators indirectly affect carbon cycling by altering microbial densities and community composition and can increase CUE by alleviating competition among microbes. Microbes are consumed by carnivorous organisms, including other microbes and soil animals, such as nematodes, arthropods, and soil vertebrates [64]. Microbes are also consumed by omnivorous organisms, such as earthworms and springtails, which feed on living microbes and microbial detritus [64]. A seminal study on protozoan predation found that predators at high grazing intensity increased the CUE of the soil community [65]. Despite such findings, surprisingly little work has followed up on these results, so it remains unclear how effects of predation on microbial CUE vary across environmental conditions and involving different types of grazers and prey.

Predator density has well-known effects on microbial processes related to CUE, such as respiration, decomposition and growth [16, 66]. For example, a study on earthworm invasions in two deciduous forests showed that earthworms reduced microbial biomass by 42% and soil respiration by 32%, potentially leading to decreased CUE via increased water stress induced by earthworms [66]. The negative effect of earthworms on respiration and microbial biomass was greatest at the edge of the invasion but had less pronounced effects in areas where earthworm biomass was higher. Thus, one possibility is that high earthworm densities have a smaller effect on CUE than intermediate earthworm densities and, at least in this study, this occurs due to reduced soil moisture at the invasion edge [66]. Interestingly, in another study by [67], high predator density decreased both decomposition and respiration rates [67]. Corroborating these results, hyphal extension of soil fungi is restricted at high predator densities, whereas at low predator densities, fungal growth rates increase, thereby potentially also reducing CUE [16]. These findings collectively suggest that while any form of predation can impact microbial growth processes, CUE may be particularly reduced at low compared to high predator densities. Future work should test this hypothesis by directly measuring CUE in the context of different grazer densities.

Predators also impact the spatial organization of microbial communities by enhancing dispersal of propagules (spores, hyphae) via predator gut passages and faecal deposition, as well as through passive transport on predator exoskeletons [16, 68]. In some contexts, dispersal may enhance CUE since dispersed microbes can accidentally be deposited into less stressful or less competitive environments [69]. Indeed, many microbes produce volatile organic compounds in order to attract animals that disperse their propagules [70]. It is possible that dispersal through predation is highly stochastic, but the common observation of preferential grazing, i.e. the preference of predators for specific prey, indicates that it may be possible to predict which microbes are most likely to be consumed by predators and thus have their propagules dispersed. For instance, selective grazing for weak and strong microbial competitors has been repeatedly observed, such as collembola preferring to consume less competitive fungi [16, 68, 71]. In contrast to enhancing CUE, there is also evidence that by increasing the spatial range of prey species, predators may force microbes to forage on low quality, nutrient-limited substrates [68], thus reducing CUE. In short, the consequences of higher trophic level-mediated dispersal on CUE likely depend on context, predator preference, the life history strategy of the prey and stochastic processes. However, this has not been empirically tested, and future studies should explicitly measure microbial CUE in different prey–predator systems and in animal-based studies of dispersal.

### Effects of Plant Community and Plant–Microbe Interactions on CUE

Plant–microbe interactions have the potential to impact CUE by regulating microbial metabolism, shaping microbial community composition, as well as by physically altering the soil environment. Plants (autotrophic organisms) and soil microbes (usually heterotroph organisms) often enter into reciprocal interactions, whereby microbes consume plant root exudates, mineralize organic matter (including plant litter) and liberate nutrients for plants [72]. Different plants produce distinct profiles of exudates that select for specific microbial species in the rhizosphere [72, 73]. Microbial mutualists of plants include—but are not limited to—nitrogen-fixing bacteria, arbuscular mycorrhizal fungi and ectomycorrhizal fungi [73]. All are abundant in certain rhizospheres and can profoundly impact ecosystem functioning. Presumably, these mutualists also impact the CUE of the free-living soil microbial community via changes to nutrient availabilities and competitive interactions, and may function to lower heterotrophic microbial CUE in the rhizosphere. Nevertheless, plants also affect the CUE of their microbial symbionts and may even enhance CUE by providing soil conditions beneficial to microbial growth.

Plants, by shaping soil conditions, such as moisture content, nutrient inputs and habitat space via litter and root inputs, fuel microbial metabolism. In some contexts, plants may create conditions that enhance CUE. This is evidenced by the fact that the presence of plants in comparison to bare fallow increases soil water content [74], which tends to increase microbial CUE by reducing stress, increasing substrate diffusion and promoting cooperation among microbes [53, 75]. Further, by exuding carbon compounds, plants provide microbes with labile energy sources [73] that have the capacity to increase the CUE of carbon-limited free-living microbes. However, such a mechanism could alternatively represent a metabolic cost to nitrogen-limited microbes, reducing their CUE. Meier et al. [76] demonstrated that root exudates increase nitrogen mineralization and investment in nitrogen acquisition enzymes, increasing nitrogen availability but not microbial biomass. The amount and types of plant exudates released are also plant species-specific [75] and vary with local plant diversity, which may explain why diverse plant systems have higher microbial biomass and lower respiration rates than plant monocultures [77]. Plants may increase microbial CUE if they modify abiotic conditions that reduce stress and balance microbial stoichiometric demands, particularly if plant diversity is high; thus, plant effects are likely highly localized and context dependent.

There is also evidence for plants to reduce microbial CUE, with recent evidence showing that plants select for less efficient microbial communities in the rhizosphere compared to microbes living in bulk soil [78]. This can occur through three mechanisms. First, plants alter soil nutrient availability by competing with microbes for resources [73, 79]. For instance, Moreau et al. [79] demonstrated that the abundance of nitrate-reducing bacteria decreased as a function of plant nitrogen-use efficiency. If plants deplete bioavailable nitrogen pools, microbes must invest in more intensively into nutrient acquisition strategies in order to mine nitrogen from SOM or mineral complexes, which can reduce CUE [5]. Second, by directly altering soil nutrient availability [80], root exudates can induce microbial nitrogen limitation and lead to reduced microbial CUE of N-limited microbes [as discussed above; 5]. Finally, plants select for specific microbial species in the rhizosphere that may be either *r*-strategists [73] or K-strategists [81], which can impact CUE at the community level if *r*-strategists grow less efficiently [82]. Ultimately, by reducing nutrient availability and selecting for unique microbial communities in the rhizosphere, plants may favour the proliferation of fast-growing microbes via indirect competition and select for microbes with high investments in resource acquisition (A-strategy). This can decrease CUE at the community level, but such an effect may additionally depend on the plant species involved and environmental context of the rhizosphere.

Some plants additionally produce secondary metabolites (e.g. allelochemicals) that induce stress in microbial communities and suppress microbial growth [83]. For example, the release of arabinogalactan-proteins, jasmonic acid, salicylic acid, and flavonoids serves as important plant defence compounds that inhibit fungal growth [84], which includes pathogens that are commonly facultative saprotrophs [85]. Some plants also deploy toxic compounds to suppress symbiotic microbes that associate with plant competitors [86]. For example, *Alliaria petiolata* produces flavonoids and aliphatic glucosinolates that suppress the growth of arbuscular and ectomycorrhizal fungi [86, 87]. *Arabidopsis thaliana* produces indolic glucosinolates that strongly reduce mycorrhizal colonization [88]. Some plant species, such as *Cucumis sativus*, also exude compounds, such as the amino acid tryptophan, to enhance colonization of plant growth-promoting rhizobacterium—the presence of which suppresses other soil community members [83]. Plant growth-promoting rhizobacteria suppresses soil-borne pathogens by releasing antibiotic compounds and is also competitive for soil micronutrients, both of which could lower community CUE [83]. Plant chemicals that induce stress and reduce the growth of key components of the microbial community could have potentially substantial impacts on CUE. Even if microbes do adapt to such stressful conditions, then they have adopted an S-based life history strategy, which is associated with metabolic costs. While empirical studies of the costs induced by resistant species are needed to validate such a hypothesis, it could explain why rhizosphere microbes grow less efficiently than bulk soil microbes among certain plant species [78].

Plant–microbe symbioses may affect microbial CUE by altering interactions between symbiotic microbes and free-living microbes. Whether microbial symbionts increase or decrease community CUE depends on the ecology and physical habitat of symbionts. For example, it has been suggested that microbial endophytes, by colonizing the inside of the root, experience reduced competition against other microbes living in the rhizosphere [73]. Soil systems dominated by arbuscular mycorrhizal fungi, which are relatively weak decomposers, are associated with higher CUE than those dominated by ectomycorrhizal fungi, which have retained a high decomposing ability and are strong competitors against saprotrophic fungi [69]. Indeed, it has been shown that the CUE of ectomycorrhizal fungal mycelium across eight boreal forests ranges from less than 5% to 20% [89], which is twice as low as the average observed for free-living microbes (50%; [35]). Thus, symbionts that compete with free-living saprotrophs—such as ectomycorrhizal fungi—may reduce overall community CUE, whereas those that physically avoid competition and are not strong competitors—like arbuscular mycorrhizal fungi or rhizobacterium—may increase community CUE.

Further, we argue that there must be flexibility in the effects of symbionts on CUE via their direct growth responses to plant compounds. A suite of plant genes encode for particular compounds that establish and regulate rhizobia and mycorrhizal symbiosis [90]. For instance, strigolactone is exudated by roots of arbuscular mycorrhizal associated host plants to facilitate mycorrhizal colonization [91]. Strigolactone has been found to stimulate arbuscular mycorrhizal fungal hyphal branching [92], and it would thus be interesting to investigate its effects on CUE. It was also recently shown that the fatty acid myristate permits a free-living life cycle in a model arbuscular mycorrhizal fungus widely considered to be an obligate biotroph [93]. Via myristate uptake, arbuscular mycorrhizal fungal growth may become unlinked to host plant carbon and lipid allocation as arbuscular mycorrhizal fungi act more independently, potentially compete with free-living microbes and alter their growth modality [94]. How plants communicate with microbial symbionts can drastically affect microbial symbiont growth, but, to our knowledge, no study has yet examined how symbiosis communication impacts either population or community-based microbial CUE.

As a final point, autotroph–heterotroph interactions are also not limited to plants and include interactions among algae and some types of bacteria. For instance, lichen is a symbiosis between fungi and photoautotrophic partners (i.e. green algae, cyanobacteria) that can occur in soil and that typically shows the pattern of K-selected organisms with a slow growth and decomposition rate [95]. The ground cover of some ecosystems is covered with mats of lichen that may play important roles in affecting CUE, particularly in extreme environments where lichen are most common. Fungi comprise most of the lichen body (i.e. thallus), which provides a controlled level of sunlight and facilitates gas exchange, while the photoautotroph redistributes energy-rich nutrients [96]. The lichen symbiosis confers an outstanding tolerance to desiccation, radiation and extreme temperature—an adaptation that may lower the cost of coping with stress in hostile habitats, including compacted soil or desert [96]. In addition to being K-selected, lichens are therefore highly stress tolerant. Further, the mass of lichen is up to 30% secondary metabolites, mostly of fungal origin [97]. High stress tolerance and substantial investment in secondary metabolites versus biomass production together suggest that lichen may be an example of facilitation that reduces the overall CUE of a system. However, this is a highly understudied area of research, and its importance in typical mineral soils that usually support low lichen populations remains unknown.

### Effect of Spatial Heterogeneity on CUE

We argue that detailed consideration should now be given to the impact of abiotic factors on biotic interactions and how this, in turn, affects CUE. For instance, soil spatial distribution affects how water, gases, nutrients, organic matter and microorganisms move through soil and in turn interact to affect CUE. Soil pore networks are especially important regulators of effective distances between species with implications for species interactions. For example, in well-connected soils with fine-particle size fractions, the rod-forming bacteria *Bacillus* out-competed the filament-forming bacteria *Streptomyces* due its faster growth rate, but in poorly-connected soils, *Streptomyces* out-competed *Bacillus* because of its ability to produce hyphae and exploit far-off resources [98]. The development of 30–150 μm pores in soil promotes connectivity and microbial enzyme activities which enhance decomposition [99]. Connectivity may reduce CUE by promoting faster microbial growth and increasing resource acquisition, both of which can constrain CUE, as discussed in this review. However, under generally anoxic conditions, the development of 30–150 μm pores promotes aerobic conditions and may increase CUE [100]. Further, pores of this size can transport molecules into active bindings sites that stabilize soil carbon [99]. Thus, even if greater connectivity reduces CUE, the positive effects of pore formation on carbon stabilization may outweigh reductions in CUE, but this remains an open question.

Soil structure also affects physical access by different sized soil organisms and protection via the exclusion of larger organisms. As an example, the survival of rhizobia was higher in soil with pores smaller versus larger than 6 µm because these small pores protected rhizobia from protozoan grazing [101]. Ritz and Young (2004) [102] proposed that fungi inhabiting soil pores smaller than the body sizes of grazers experience protection. Depending on fungal biomass and carrying capacity, this could enhance the prey’s CUE if biomass is below carrying capacity by protecting fungi from grazing or reduce CUE at carrying capacity by promoting intraspecific competition and rapid growth (see chapter 1.5). Further, Six et al. [103] suggest that pore space could reduce carbon and nitrogen decomposition by offering protection of fungi and protozoa from nematodes predation, thereby stabilizing carbon and increasing the system’s CUE.

Lastly, soil spatial distribution also includes soil biofilms serving as microhabitats for interacting species. Often microbes produce biofilms cooperatively, generating favourable conditions for efficient growth [104]. For example, diverse bacterial species in biofilms are often auxotrophic and rely on the cross-exchange of different amino acids and vitamins in order to grow [105]. Mixed species biofilms also reduce stress more than single-species biofilms by promoting tolerance to anti-microbial compounds [106]. Collectively, this suggests that biofilms create favourable spatial habitats for high CUE. However, there are also contexts in which biofilms promote competition and may reduce CUE. Notably, competition is promoted within biofilms if species are more closely related or have generalist metabolic strategies [107] and when labile resource availability is high [61]. Soil spatial distribution is strongly impacted by microbial biofilms that provide key habitats for species interactions affecting CUE. Much of this work is based on simple mesocosm studies and models and has not been explicitly linked to CUE. Tracking the role of soil spatial distributions on CUE in actual soils is an important area of future research.

## Conclusion and Further Considerations

Biotic interactions influence CUE by affecting the allocation of carbon to different metabolic processes, changing environmental conditions, and inducing shifts in microbial community composition. However, a huge variety of biotic interactions take place in soil, making it challenging to predict CUE without having a complete picture of their cumulative impacts. While we broadly show that competition is a “negative” interaction that reduces CUE, we also present evidence that indirect competition can, in some cases, positively impact CUE. At the same time, facilitation is a “positive” interaction that is generally expected to increase CUE, especially when microbial population densities are low. Although plant–microbe interactions are often facilitative, such interactions can reduce CUE in the rhizosphere and potentially more widely in ectomycorrhizal fungal-dominated systems. Furthermore, we know that biotic interactions can have complex cascading effects, with some interactions generating new interactions (see Fig. 2). For example, indirect competition can increase the probability of direct competition and facilitation can decrease the probability of direct competition or predation. Importantly, cascading effects of biotic interactions are widely known to introduce apparent stochasticity to microbial communities, which may be difficult to predict [108]. Future research must ultimately focus on teasing such apparent complexity apart.

In conclusion, by reviewing literature spanning the fields of microbiology, evolution, and botany, we analysed how soil microbial CUE is affected by a key set of biotic interactions. This is important because while there is a concerted effort to understand the abiotic factors regulating microbial CUE, surprisingly few studies have addressed the additional role of biotic interactions in soil ecosystems. Of course, studying soil microbial interactions comes with substantial technical challenges. Several methods are used to measure CUE and they differ in meaningful ways depending on whether the CUE is measured at the population, community or ecosystem scale, or in cultures, mesocosms, or actual field soil (see [109]). Creating generalizable CUE frameworks is therefore challenging when working across different scales and media. While much of the evidence presented here considering biotic interactions and CUE yields more questions than answers, this review was written to organize a path forwards. By summarizing the key theoretical biotic interactions that should affect CUE and pairing this with available supporting evidence, we were able to provide concrete research suggestions for the future. Despite addressing a new area of CUE research, we argue that understanding how biotic interactions shape microbial CUE is important not only for conceptually, but also for managing natural systems, such as to identify strategies in agricultural soils that favour biotic interactions known to increase CUE (see [43]). In our opinion, considering biotic interactions alongside abiotic drivers of CUE will ultimately improve both mechanistic insights and predictive power in ecosystem ecology and management.

## Data Availability

Not applicable.
